# Recurrent Cardiac Tamponade Following an Open Mitral Valve Replacement

**DOI:** 10.7759/cureus.116114

**Published:** 2026-09-11

**Authors:** Justin N Miranda, FNU Swatisikta, Nabir Babbar

**Affiliations:** 1 Department of Internal Medicine, HCA Florida Westside Hospital, Plantation, USA; 2 Department of Critical Care Medicine, HCA Florida Westside Hospital, Plantation, USA

**Keywords:** cardiac tamponade, cardiothoracic surgery, critical care cardiology, echocardiogram, pericardial effusion, transthoracic echocardiogram

## Abstract

Cardiac tamponade is a life-threatening complication characterized by the accumulation of fluid or blood within the pericardial sac surrounding the heart, leading to increased intra-pericardial pressure and impaired ventricular filling. This is a known complication of cardiothoracic procedures, including coronary artery bypass grafting (CABG) and mitral valve repairs, and can rapidly progress to decreased cardiac output, hypotension, and eventual cardiac collapse. We report the case of a 62-year-old male with an unremarkable past medical history who was admitted to the hospital due to recurrent fevers and bacterial infections, which were found to be due to mitral valve vegetation, and subsequently underwent open mitral valve replacement. His postoperative course was complicated by recurrent cardiac tamponade requiring multiple operative interventions, ultimately resulting in an extended ICU stay, but he was eventually discharged to inpatient rehab two weeks after the fact. The recurrent hemopericardium in this case was most consistent with surgical and coagulopathic bleeding, with a possible secondary contribution from an upregulated inflammatory response related to the patient's infective endocarditis. After any cardiac surgery, a high index of suspicion for cardiac tamponade should be maintained in patients who become hemodynamically unstable. Presentation is often nonspecific, and diagnosis requires a high index of suspicion combined with prompt imaging, with the aid of interdisciplinary collaboration in order to successfully treat patients expeditiously.

## Introduction

Cardiac tamponade is a common complication arising from open-heart surgical procedures, including coronary artery bypass grafting (CABG) and mitral valve repairs. This complication is characterized by the accumulation of fluid or blood in the pericardial sac surrounding the heart [1]. This accumulation of fluid exerts excessive external pressure on the heart, impeding its ability to pump blood to the body effectively. Early detection and treatment of cardiac tamponade is crucial in the immediate management of patients post-cardiac surgery. As a result of mitral valve surgery, bleeding into the pericardial space may occur, which could progress to life-threatening cardiac dysfunction and must be treated quickly to prevent further deterioration and irreversible damage [2]. Consequently, vigilant monitoring is essential for patients undergoing mitral valve repair to detect this potentially life-threatening complication, amongst others.

Hemodynamically relevant effusion resulting from cardiac surgery can be categorized into two groups based on the timing of symptomatic presentation. Acute cardiac tamponade is defined as the clinical presentation of tamponade within the first 48-72 hrs. Subacute or late cardiac tamponade can develop without obvious clinical signs of tamponade around three or more days after cardiac surgery. Studies have indicated that the incidence of cardiac tamponade necessitating surgical re-intervention following cardiac surgery can be as high as 6% of cases [1].

Certain risk factors may heighten the likelihood of postoperative tamponade, such as coagulopathy or increased intraoperative bleeding. Early detection and management of cardiac tamponade is crucial in preventing irreversible cardiac damage. Because there may be atypical features associated with this condition, studies have evaluated various clinical and hemodynamic signs involved with the pathophysiology of postoperative cardiac tamponade. Retrospective studies have shown that roughly 24% of patients had hypotension and hemodynamic instability, about 34% of patients suffered from right atrial collapse, and 65% had left ventricular diastolic collapse [3].

While recurrent postoperative tamponade after open cardiac surgery is a recognized complication, this case highlights the compounding effect of active infective endocarditis, with worsening tissue friability and coagulopathy, on bleeding risk after valve replacement. This case illustrates a staged management approach that involved repeat clot evacuation, a pericardial window, and delayed sternal closure with a vacuum dressing that allowed for hemodynamic stabilization while the underlying bleeding source resolved.

A version of this article was previously posted to the Authorea preprint server on September 9th, 2025; however, it is not under consideration at any journal for publication at this time.

## Case presentation

A 62-year-old male with an unremarkable past medical history was transferred to our hospital due to persistent, unremitting fevers over several weeks, along with blood cultures for *Streptococcus oralis* and *Streptococcus mitis* bacteremia. Prior to admission, the patient was not taking any antiplatelet or anticoagulant medication; however, during his stay in the hospital, he was started on a prophylactic dose of daily subcutaneous low-molecular-weight heparin. Upon admission, a transthoracic echocardiogram revealed the presence of a 1.6 cm mitral valve vegetation and concerns for infective endocarditis (Figure 1). Upon arrival to the hospital, his vital signs were as follows: heart rate 107 beats per minute, blood pressure 133/86 mmHg, respiratory rate of 20 breaths per minute, pulse oximetry of 98%, and temperature of 103.1 degrees Fahrenheit (Table 1). 

**Figure 1:**
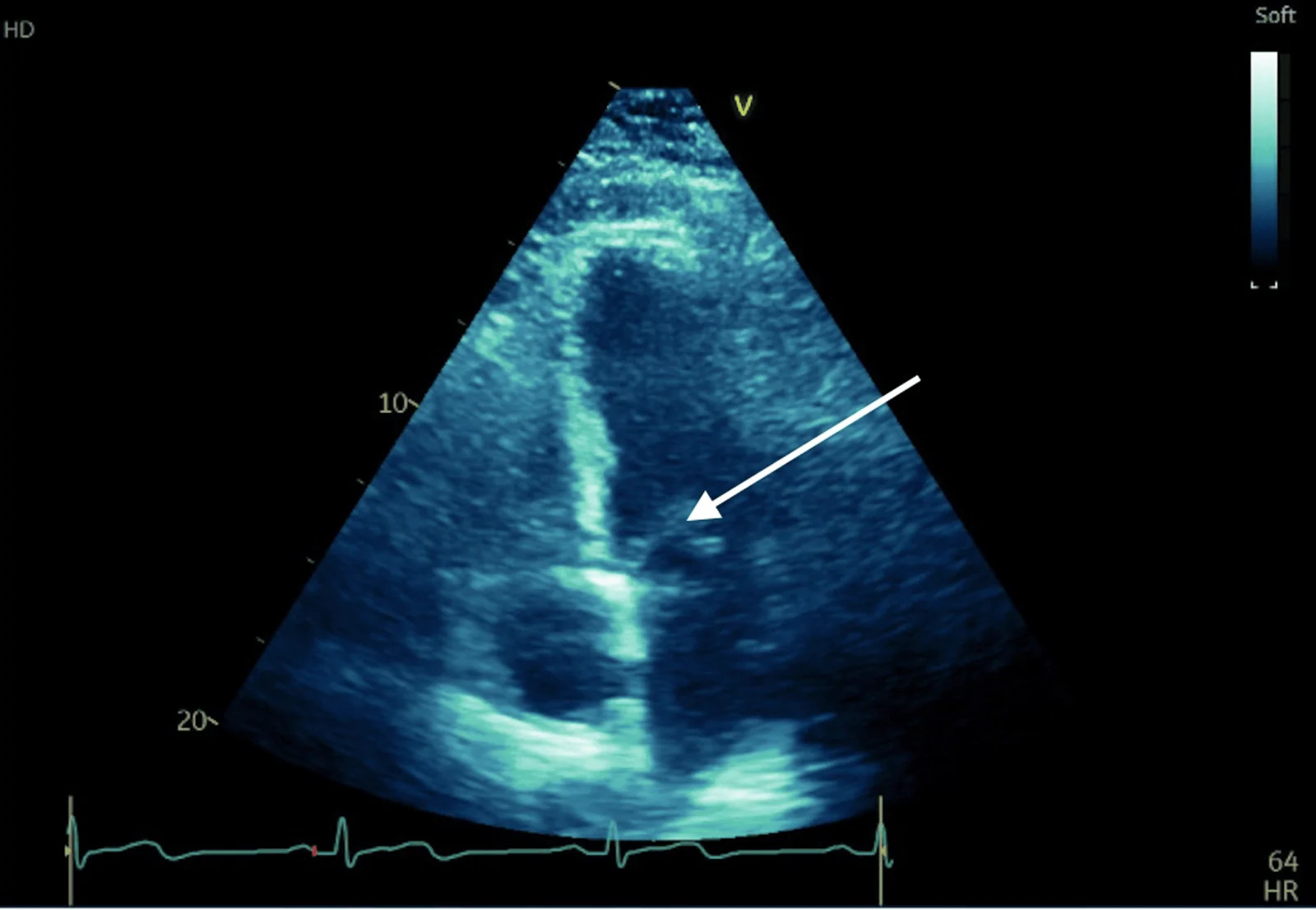
Bedside transthoracic echocardiogram indicating a mobile mass in the mitral valve, representative of mitral valve vegetation. The arrow is pointing at the mobile mass on the mitral valve leaflet.

**Table 1:** Patient vital signs upon admission, post-op (MVR) Day 0 and Day 7, compared to standard reference vital signs for a normal, healthy patient. MVR: mitral valve repair

Vitals	Heart Rate (BPM)	Systolic Blood Pressure (mmHg)	Diastolic Blood Pressure (mmHg)	Respiratory Rate (breaths per min)	Pulse Oximetry (%)	Temperature (Degrees Fahrenheit)
Vital Signs on Admission	107	133	86	20	98	103.1
Vital Signs Post-op Day 0	82	110	70	14	100	98.1
Vital Signs Post-op Day 7	101	105	60-80	18	98	98.6
Reference Vital Signs	60-100	90-120	60-80	12-18	95-100	97.8-99.1

Subsequently, the patient underwent a procedure for a mitral valve replacement with a bioprosthetic prosthesis and a left atrial appendage clip (standard protocol for this cardiothoracic surgeon), following which he was transferred to the Intensive Care Unit (ICU) for further observation and management. During the surgery, the patient received three liters of crystalloids, 2000 units of prothrombin complex concentrate, one unit of packed red blood cells, and two units of platelets. Throughout the surgery, the non-surgical diffuse oozing was observed from all tissue surfaces, prompting empiric correction of coagulopathy. Intraoperative international normalized ratio (INR) was 1.1, hemoglobin was 12.5 g/dL, and platelets were 144 uL (Table 2). Postoperatively, the patient was transferred to the ICU with mediastinal, pericardial, and right pleural chest tubes in place. No epicardial pacing wires were placed at the time of surgery.

**Table 2:** Laboratory values. Pertinent laboratory values intraoperatively and on post-op Day 9, when decompensation occurred, with reference values. INR: international normalized ratio; AST: aspartate aminotransferase; ALT: alanine aminotransferase

Lab Values	Hemoglobin (g/dL)	Platelets (µL)	INR	Creatinine (mg/dL)	AST (units/L)	ALT (units/L)
Intraop Labs	12.5	144,000	1.1	0.89	35	27
Labs Post-op Day 9	13.4	205,000	1.2	3.49	3643	3041
Reference Lab Values	13.5-17.5	150,000-450,000	0.8-1.1	0.7-1.3	8-40	7-56

His initial ICU admission was rather uneventful, with the only notable concern being mild, persistent low-grade fevers, despite continuous intravenous antibiotic administration. During this time, he denied any shortness of breath or chest pain. On postoperative Day 7, he was discharged from the critical care team’s care and was transferred to a post-critical care unit, hemodynamically stable on antibiotics and goal-directed medical therapy. A final CT scan performed on his last day in the ICU revealed the absence of a pulmonary embolism, the presence of a small stable retrosternal hematoma, and small bilateral pleural effusions. Coagulation studies were not obtained at this time, as the patient was hemodynamically stable and bleeding was not believed to be clinically significant.

Two days after transfer from the ICU, a rapid response was triggered due to profound hypotension, tachycardia, oliguria, and a more somnolent mental status, with laboratory evaluation notable for profound acute renal failure and hepatic dysfunction. Rapid labs were notable for a creatinine of 3.49 mg/dL, aspartate aminotransferase (AST) of 3643 units/L, and alanine aminotransferase (ALT) of 3041 units/L (Table 2). A transthoracic echocardiogram was performed at bedside (Figure 2), which showed an obvious large collection of pericardial fluid surrounding the right and left ventricles. He was urgently taken to the OR for a pericardial window and evacuation. Remarkably, the patient tolerated the procedure well without any complications or hemodynamic instability. Approximately 250 mL of sanguinous fluid was removed from the pericardial cavity, with an additional 550 mL of serosanguineous fluid removed from the right pleural space. A postoperative transesophageal echocardiogram demonstrated a normal ejection fraction and normal valvular function. Given the clinical circumstances, the patient was subsequently intubated and readmitted to the ICU, with chest tubes in place, to ensure ongoing monitoring and therapeutic management. He was transferred to the ICU once again, intubated and with chest tubes in place. At this time, all anticoagulation medication (including deep vein thrombosis (DVT) prophylactic doses) was stopped.

**Figure 2:**
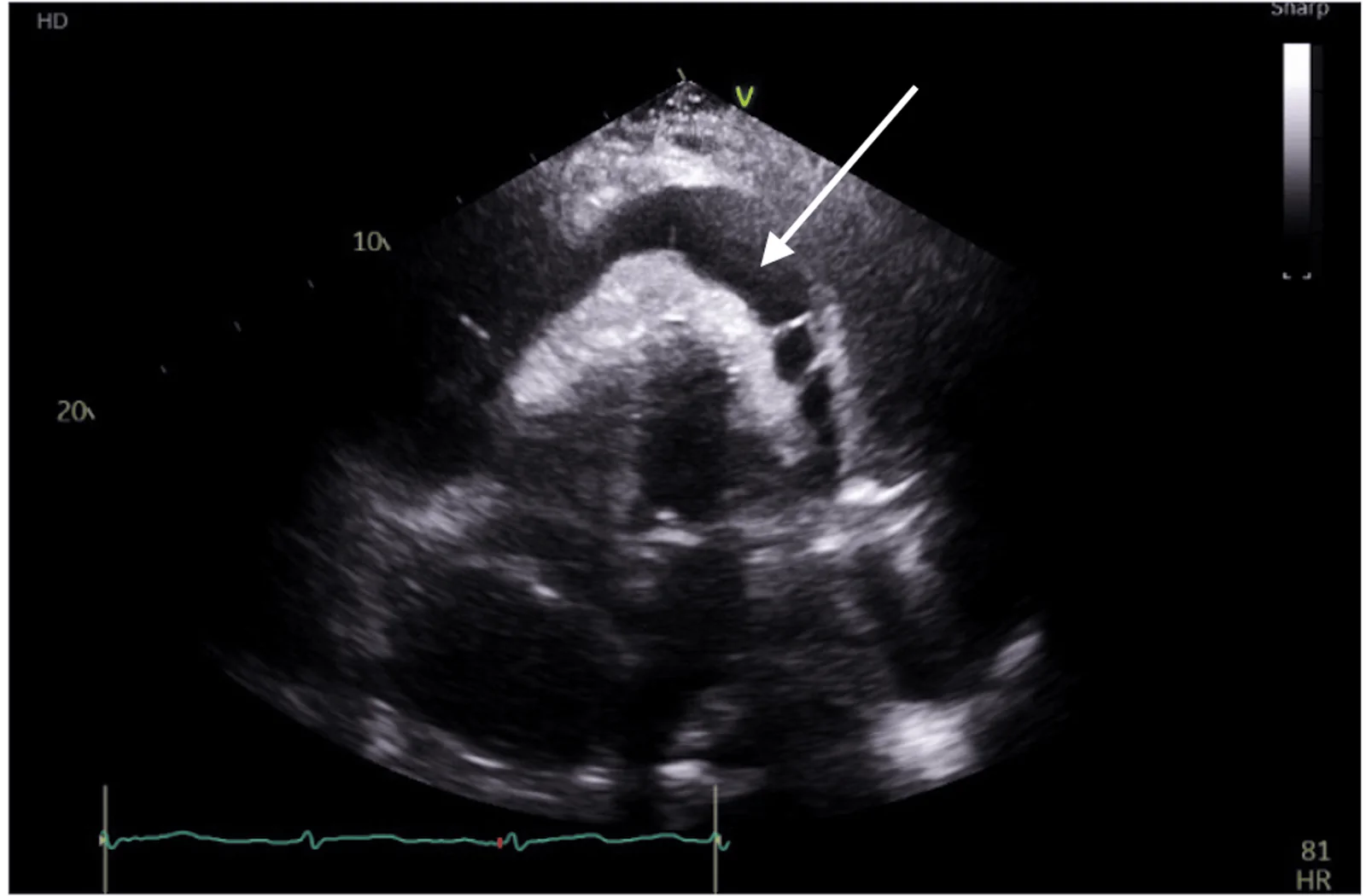
Transthoracic echocardiogram, parasternal long-axis view, demonstrating a large circumferential pericardial effusion surrounding the heart. The arrow is pointing at the fluid collection surrounding the pericardial sac.

The next day, a CT scan of the chest was noted for an increasing size of a large multi-loculated hemopericardium with the largest component compressing the right atrium, measuring a maximal thickness of 5.3 cm (Figure 3). The patient subsequently decompensated in the ICU; repeat imaging indicated an increasing cardiac silhouette and worsening hematoma and cardiac tamponade. He was once again urgently sent to the OR for repeat clot evacuation, and the chest was left open with a wound vac in place. Two days after this procedure, the patient was sent to the OR one last time for chest closure. The procedure was completed without complications, and he was sent back to the ICU, intubated and hemodynamically stable.

**Figure 3:**
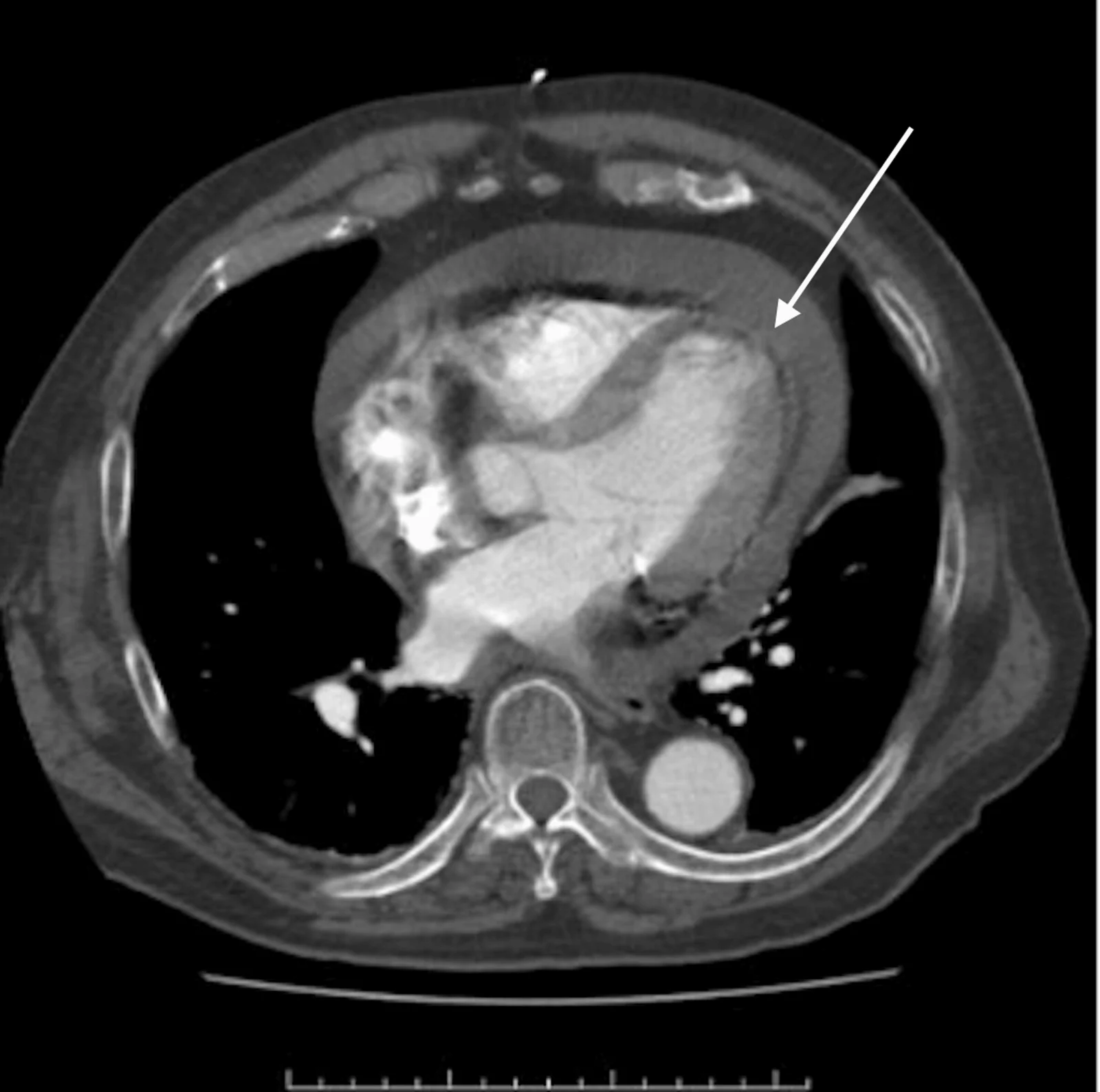
CT scan of the chest of a large multi-loculated hemopericardium. The arrow is pointing to fluid surrounding the heart within the pericardium, seen on CT scan.

Outcome and follow-up

About 12 days after the patient’s initial mitral valve surgery, he returned to the ICU following his final procedure. This last procedure was a sternal closure after having repeated hematoma evacuations from a recurring cardiac tamponade. After the surgical closure, he remained in the ICU for close monitoring for three nights. During this time, he remained hemodynamically stable, not requiring any vasopressors or significant amounts of crystalloid or blood products. At this time, there was complete resolution of renal dysfunction, and liver enzymes returned to baseline with adequate fluid resuscitation and resolution of cardiac tamponade physiology. All in all, he spent about 13 days in the ICU, along with three major cardiac procedures; after this time, the patient returned to the floor, where he spent a significant portion of his time with the physical therapy team getting his strength back. Once the patient was stable and ready for discharge, arrangements were made to send him to a rehab center to help maintain his physical health in the immediate future. At the time of discharge, he was able to walk by himself, perform daily tasks, and was completing a course of intravenous antibiotics.

There are currently no set guidelines regarding the surveillance and follow-up requirements in patients who have suffered from cardiac tamponade. At this time, the only recommendation consists of close follow-up with a trained cardiologist after the hospital stay. Follow-up exams would include serial physical exams, follow-up echocardiograms, and EKGs. It may be indicated to follow up with transesophageal echocardiograms if the patient simultaneously suffered from any valvular disease. This would help monitor any future vegetation growth, concerning for repeat infection and a high likelihood of tamponade recurrence.

## Discussion

After cardiac surgery, the incidence of cardiac tamponade may vary by surgery type and precipitating factors throughout the course of the case. For patients undergoing coronary artery bypass graft surgery, the incidence reportedly ranges from 0.2% to 8.4% [4]. Certain factors can play a role in determining the risk of cardiac tamponade postoperatively. These factors include the administration of anticoagulants either intraoperatively or preoperatively, intraoperative mediastinal bleeding, the removal of epicardial pacing wires, the presence of autoimmune disorders, or even the presence of hematological conditions [4]. The signs of cardiac tamponade can be very nonspecific, and there may be a degree of difference patient by patient. A high degree of suspicion is all that is necessary, with the addition of clinical examination, to establish the diagnosis. Due to the impending cardiopulmonary collapse, the deployment of imaging techniques must always be expedited in order to avoid any delays in diagnosis and the initiation of intervention. Delayed cardiac tamponade, which occurs three days postoperatively, is recognized as a notable and detrimental complication following cardiac surgery, carrying a higher mortality risk compared to earlier diagnosed tamponade. In those patients who experience delayed pericardial effusion diagnosis postoperatively, the 30-day mortality rate was reported to be 4% [5].

In all forms of cardiac tamponade, the pericardial sac gradually stretches to accommodate the increased fluid volume. During a normal cardiac cycle, the total intrapericardial volume remains relatively constant. However, if the pericardium’s stretching capabilities are overwhelmed by a rapid influx of fluid, any additional fluid can lead to elevated pressure within the pericardial sac. As this intrapericardial pressure exceeds that of the intracardiac pressure, a positive transmural pressure gradient can develop, which leads to compression of cardiac chambers. Because the nature of intracardiac pressures is to fluctuate throughout the cardiac cycle, the pericardial pressure may exceed intracardiac pressures within different chambers at different points in the cycle. This leads to a state of hemodynamic instability, characterized by the heart’s struggle to maintain adequate blood flow in response to the changes in fluid volume status [5].

In the context of cardiac tamponade, compensatory mechanisms primarily involve adrenergic stimulation. The purpose of these mechanisms can be broken down into two categories: alpha versus beta receptors. The purpose of involving the beta adrenergic receptors would be for augmentation of the heart rate, increasing it to help increase cardiac output. Activation of these receptors also helps with the enhancement of diastolic relaxation to allow for more cardiac filling to help optimize cardiac output. Lastly, beta receptor activation aids in the amplification of inotropy to facilitate an increase in the ejection fraction. The involvement of alpha adrenergic receptors in the setting of tamponade would be to elevate peripheral arterial resistance to ensure sufficient coronary blood flow [6].

The clinical manifestations of postoperative cardiac tamponade encompass a wide array of symptoms and clinical signs. Some of the more common and specific signs include dyspnea, diminished cardiac sounds, chest pain, orthopnea, and even cardiogenic shock. The presence of hemodynamic instability characterized by tachycardia and hypotension is a specific finding present in cardiac tamponade, regardless of the precipitating factors. The emergence of pulsus paradoxus represents a critical clinical indicator that suggests the presence of cardiac tamponade. Notably, this finding may not be present in postoperative patients undergoing mechanical ventilation or with the presence of certain comorbidities. Therefore, respiratory variability observed in the pulse oximetry waveform can serve as an indirect indicator of hemodynamic compromise in spontaneously breathing patients who are at risk for pericardial effusion or tamponade [7].

The diagnosis of tamponade may also be concluded with the help of diagnostic imaging. On a chest X-ray, however, a normal heart size may be seen despite clinical signs and symptoms of cardiac tamponade. Once the effusion volume reaches a quantity of about 200 mL, then the cardiac silhouette will appear enlarged compared to normal on a chest X-ray, taking the appearance of a water bottle [7]. Importantly, the only electrocardiographic sign leading towards the diagnosis of tamponade is the presence of electrical alternans, which is a variation in the contour and height of QRS complexes for each beat. On echocardiography, accurately assessing the presence and size of pericardial effusions is crucial for quantifying hemodynamic consequences and guiding treatment strategies. Some signs on echocardiogram include compression of the right atrium during early diastole, compression of the right ventricle during late diastole, and collapse of both chambers during deep expiration. You may also see a delay of mitral valve opening during inspiration, which may suggest deficits in left ventricular filling. Collapse of the right atrium is suggestive for diagnosis in tamponade; the collapse of the right ventricle is more specific for diagnosis [7].

In this case, the character and timing of the fluid collections are most consistent with recurrent hemorrhage rather than an inflammatory process. The pericardial fluid remained grossly serosanguineous at each intervention, despite the known bacteremia and valvular endocarditis. The patient's active infective endocarditis and associated coagulopathy, together with the intraoperative diffuse non-surgical oozing, make surgical and coagulopathic bleeding the most probable explanation for the recurrent hemopericardium. Several additional clinical features further support a surgical rather than inflammatory cause of the bleeding. Throughout the hospital stay, the patient continued to receive appropriate intravenous antibiotic therapy, remained afebrile, and his white blood cell count remained within normal limits. These findings argue against an active infectious or inflammatory process as the primary driver of fluid reaccumulation. An inflammatory contribution to fluid accumulation from an upregulated systemic inflammatory state cannot be entirely excluded, given the patient's underlying bacteremia and infective endocarditis. However, fluid reaccumulation in this case should be considered a secondary rather than primary mechanism, absent any clinical markers denoting active inflammation, leukocytosis, or fever, and given that pericardial fluid was never sent for culture or analysis. Surgical and coagulopathic hemorrhage remain the most likely explanation for recurrent hemopericardium, though an inflammatory component should be in the differential diagnosis in similar future cases. 

## Conclusions

After any cardiac surgery, a high index of suspicion for the occurrence of life-threatening situations, such as cardiac tamponade, should be maintained. Pericardial tamponade should always be considered as a complication of cardiac surgery, even when symptoms are atypical. As the presentation can be nonspecific and the time window to life-threatening deterioration can be very short, interdisciplinary collaboration is often imperative to successfully treat patients immediately. This is all the more important for patients with infective endocarditis or other conditions when the systemic inflammatory response is expected to be unregulated preoperatively. It could be an infection or an autoimmune condition.

The reliance on classic clinical findings such as Beck's triad, while important, is insufficient, as their absence does not exclude tamponade; echocardiography should remain the primary diagnostic tool. Further use of imaging studies such as a point-of-care echocardiogram or transesophageal echocardiography is helpful in detecting early signs of pericardial effusion, allowing for intervention before tamponade. This case suggests that routine surveillance echocardiography in high-risk postoperative patients could be instrumental in early detection, monitoring progression, and helping to guide treatment. Implementing clear protocols and specific algorithms for diagnosing, monitoring, and treating postoperative cardiac tamponade is crucial. This includes rapid access to pericardiocentesis or surgical exploration, as well as risk stratification. Identifying high-risk patients preoperatively and employing preventative measures such as baseline echocardiograms coupled with vigilant postoperative care could improve management; retrospectively, these are protocols that we could have improved.

## References

[1] Chuttani K, Tischler MD, Pandian NG, Lee RT, Mohanty PK (1994). Diagnosis of cardiac tamponade after cardiac surgery: Relative value of clinical, echocardiographic, and hemodynamic signs. AHJ.

[2] Harskamp RE, Meuzelaar JJ (2010). Recurrent late cardiac tamponade following cardiac surgery: a deceiving and potentially lethal complication. Acta Cardiol.

[3] Leiva EH, Carreño M, Bucheli FR, Bonfanti AC, Umaña JP, Dennis RJ (2018). Factors associated with delayed cardiac tamponade after cardiac surgery. Ann Card Anaesth.

[4] Stefan van Dinter, Li W, Wollersheim L (20231). Variations in current clinical practice of postoperative pericardial effusion: a questionnaire study. Open Heart.

[5] Yuriditsky E, Horowitz JM (2024). The physiology of cardiac tamponade and implications for patient management. J Crit Care.

[6] Cattadori G, Picozzi A, Tagliabue E (2026). Pericardial effusion after cardiac surgery: prevalence, characteristics, risk factors and management. J Clin Med.

[7] Carmona P, Mateo E, Casanovas I (2012). Management of cardiac tamponade after cardiac surgery. JCVA.

